# Dibut­yl[*N*-(5-chloro-2-oxidobenzyl­idene)-l-isoleucinato-κ^3^ *O*,*N*,*O*′]tin(IV)

**DOI:** 10.1107/S1600536809039245

**Published:** 2009-10-03

**Authors:** Hong-Jun Yang, Yan-Qiu Dang

**Affiliations:** a Research Center for Eco-Environmental Sciences Yellow River Delta, Binzhou University, Binzhou 256600, People’s Republic of China; b Department of Chemistry & Chemical Engineering, Binzhou University, Binzhou 256600, People’s Republic of China

## Abstract

The Sn^IV^ atom of the title compound, [Sn(C_4_H_9_)_2_(C_13_H_14_ClNO_3_)], adopts a distorted SnNC_2_O_2_ trigonal-bipyramidal geometry with a mean Sn—C distance of 2.105 Å and with Sn—O = 2.107 Å, and forms five- and six-membered chelate rings with the tridentate ligand. One butyl group is disordered over two positions with site occupancies of 0.65 (1):0.35 (1).

## Related literature

For the structures and biological activity of diorganotin complexes with Schiff bases derived from α-amino acids, see: Baul *et al.* (2007 ▶); Beltran *et al.* (2003 ▶); Dakternieks *et al.* (1998 ▶); Tian *et al.* (2004 ▶, 2006 ▶, 2007 ▶, 2009 ▶).
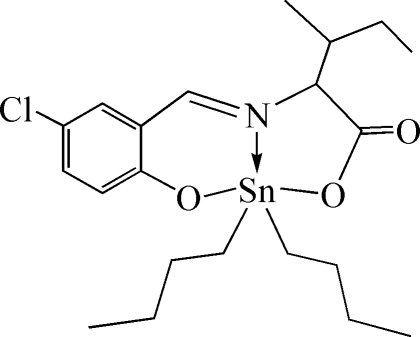


## Experimental

### 

#### Crystal data

[Sn(C_4_H_9_)_2_(C_13_H_14_ClNO_3_)]
*M* *_r_* = 500.62
Orthorhombic, 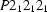

*a* = 10.0545 (14) Å
*b* = 14.497 (2) Å
*c* = 15.953 (2) Å
*V* = 2325.3 (5) Å^3^
*Z* = 4
Mo *K*α radiation
μ = 1.23 mm^−1^
*T* = 295 K
0.35 × 0.22 × 0.08 mm

#### Data collection

Bruker SMART APEX area-detector diffractometer
Absorption correction: multi-scan (*SADABS*; Bruker, 2002 ▶) *T*_min_ = 0.672, *T*_max_ = 0.908
18112 measured reflections
4574 independent reflections
3846 reflections with *I* > 2σ(*I*)
*R*_int_ = 0.036

#### Refinement

*R*[*F* ^2^ > 2σ(*F* ^2^)] = 0.040
*wR*(*F* ^2^) = 0.097
*S* = 1.05
4574 reflections
257 parameters
40 restraints
H-atom parameters constrained
Δρ_max_ = 0.51 e Å^−3^
Δρ_min_ = −0.54 e Å^−3^
Absolute structure: Flack (1983 ▶), 1978 Friedel pairs
Flack parameter: 0.00 (4)

### 

Data collection: *SMART* (Bruker, 2002 ▶); cell refinement: *SAINT* (Bruker, 2002 ▶); data reduction: *SAINT*; program(s) used to solve structure: *SHELXS97* (Sheldrick, 2008 ▶); program(s) used to refine structure: *SHELXL97* (Sheldrick, 2008 ▶); molecular graphics: *ORTEP-3 for Windows* (Farrugia, 1997 ▶); software used to prepare material for publication: *SHELXL97*.

## Supplementary Material

Crystal structure: contains datablocks global, I. DOI: 10.1107/S1600536809039245/hg2563sup1.cif

Structure factors: contains datablocks I. DOI: 10.1107/S1600536809039245/hg2563Isup2.hkl

Additional supplementary materials:  crystallographic information; 3D view; checkCIF report

## Figures and Tables

**Table 1:** Selected bond lengths (Å)

Sn1—O1	2.083 (3)
Sn1—C14	2.100 (6)
Sn1—C18	2.110 (6)
Sn1—O2	2.130 (3)
Sn1—N1	2.169 (3)

**Fig. 1.**
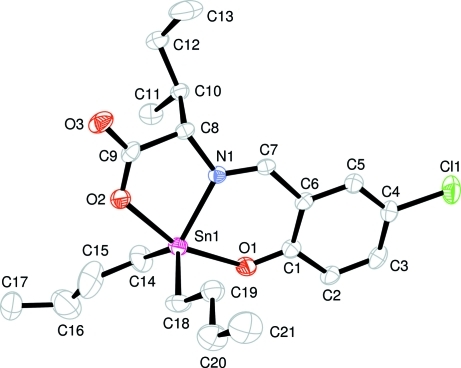
The structure of (I) with displacement ellipsoids are drawn at the 30% probability level. The minor disordered component of C14—C17 atoms of the butyl group and the H atoms have been omitted for clarity.

## supplementary crystallographic information

### Comment

The diorganotin complexes with Schiff bases derived from α-amino acids continue to received attention because of their structural diversity and biological activities (Beltran *et al.*, 2003; Basu Baul *et al.*, 2007; Dakternieks *et al.*, 1998; Tian *et al.*, 2004, 2006, 2007, 2009). The structures of the diorganotin complexes based on the Schiff base ligand [*N*-(2-hydroxyphenylmethylene)isoleucine, [*N*-(2-oxidophenylmethylene)isoleucinato]dibutyltin (Beltran *et al.*, 2003) and [*N*-(5-chloro-2-oxidophenylmethylene)isoleucinato]dicyclohexyltin (Tian *et al.*, 2004) have been reported. As a continuation of these studies, the structure of the title compound, (I), is here described.

The coordination geometry of the tin atom in (I) is that of a distorted trigonal bipyramid with two butyl groups (C14 and C17) and the imino N1 atom occupying the equatorial positions and the axial positions being occupied by a unidentate carboxylate O2 atom and a phenoxide O1 atom (Fig. 1). The tin atom is 0.033 (3) Å out of the NC2 trigonal plane in the direction of the O1 atom. The bond length of Sn1—O1 (2.083 (6) Å) was shorter than that of Sn1—O2 (2.130 (3) Å). The bond angle O1—Sn1—O2 was 158.65 (13) °, which is slightly larger than that found in [*N*-(2-oxidophenylmethylene)isoleucinato]dibutyltin (154.5 (3)°) (Beltran *et al.*, 2003) and [*N*-(5-chloro-2-oxidophenylmethylene)isoleucinato]dicyclohexyltin (153.84 (12)°) (Tian *et al.*, 2004). Distortions from the ideal geometry may be rationalized partly by the restricted bite angles of the tridentate ligand. Neither of the five or six-membered rings formed upon chelation are planar, as seen in the following torsion angles: Sn1—O2—C9—C8 9.5 (6)°, Sn1—N1—C8—C9 13.4 (4)°, Sn1—O1—C1—C6 - 14.7 (7)° and Sn1—N1—C7—C6 10.4 (7)°.

### Experimental

The title compound was prepared by the reaction of dibutyltin oxide (0.498 g, 2 mmol), L-isoleucine (0.262 g, 2 mmol) and 5-chlorosalicylaldehyde (0.314 g, 2 mmol) in 60 ml of benzene were refluxed for 8 h with azeotropic removal of water *via* a Dean-Stark trap. The resulting clear solution was evaporated under vacuum and the yellow crystalline material obtained was recrystallized from methanol. The product (yield 68%, m.p. 378–379 K) was then dissolved in dichloromethane-hexane (1:1, V/V), and yellow crystals were grown by slow evaporation.

### Refinement

A butyl group (C14—C17) is disordered over two positions. Site occupancy factors were refinded to 0.65 (1) for atoms C14—C17 and 0.35 (1) for atoms C14'-C17'. H atoms were placed at calculated positions (C—H = 0.93–0.98 Å) and refined as riding with *U*_iso_(H) = 1.2*U*_eq_(C) or *U*_iso_(H) = 1.5*U*_eq_(methyl C).

### Crystal data

**Table:**

[Sn(C_4_H_9_)_2_(C_13_H_14_ClNO_3_)]	*F*(000) = 1024
*M**_r_* = 500.62	*D*_x_ = 1.430 Mg m^−^^3^
Orthorhombic, *P*2_1_2_1_2_1_	Mo *K*α radiation, λ = 0.71073 Å
Hall symbol: P 2ac 2ab	Cell parameters from 5601 reflections
*a* = 10.0545 (14) Å	θ = 2.4–23.1°
*b* = 14.497 (2) Å	µ = 1.23 mm^−^^1^
*c* = 15.953 (2) Å	*T* = 295 K
*V* = 2325.3 (5) Å^3^	Block, yellow
*Z* = 4	0.35 × 0.22 × 0.08 mm

### Data collection

**Table:**

Bruker SMART APEX area-detector diffractometer	4574 independent reflections
Radiation source: fine-focus sealed tube	3846 reflections with *I* > 2σ(*I*)
graphite	*R*_int_ = 0.036
φ and ω scans	θ_max_ = 26.0°, θ_min_ = 1.9°
Absorption correction: multi-scan (*SADABS*; Bruker, 2002)	*h* = −12→12
*T*_min_ = 0.672, *T*_max_ = 0.908	*k* = −17→17
18112 measured reflections	*l* = −19→19

### Refinement

**Table:**

Refinement on *F*^2^	Secondary atom site location: difference Fourier map
Least-squares matrix: full	Hydrogen site location: inferred from neighbouring sites
*R*[*F*^2^ > 2σ(*F*^2^)] = 0.040	H-atom parameters constrained
*wR*(*F*^2^) = 0.097	*w* = 1/[σ^2^(*F*_o_^2^) + (0.0474*P*)^2^ + 0.7356*P*] where *P* = (*F*_o_^2^ + 2*F*_c_^2^)/3
*S* = 1.05	(Δ/σ)_max_ = 0.001
4574 reflections	Δρ_max_ = 0.51 e Å^−^^3^
257 parameters	Δρ_min_ = −0.54 e Å^−^^3^
40 restraints	Absolute structure: Flack (1983), 1978 Friedel pairs
Primary atom site location: structure-invariant direct methods	Flack parameter: 0.00 (4)

### Special details

**Table:**

Geometry. All e.s.d.'s (except the e.s.d. in the dihedral angle between two l.s. planes) are estimated using the full covariance matrix. The cell e.s.d.'s are taken into account individually in the estimation of e.s.d.'s in distances, angles and torsion angles; correlations between e.s.d.'s in cell parameters are only used when they are defined by crystal symmetry. An approximate (isotropic) treatment of cell e.s.d.'s is used for estimating e.s.d.'s involving l.s. planes.
Refinement. Refinement of *F*^2^ against ALL reflections. The weighted *R*-factor *wR* and goodness of fit *S* are based on *F*^2^, conventional *R*-factors *R* are based on *F*, with *F* set to zero for negative *F*^2^. The threshold expression of *F*^2^ > σ(*F*^2^) is used only for calculating *R*-factors(gt) *etc*. and is not relevant to the choice of reflections for refinement. *R*-factors based on *F*^2^ are statistically about twice as large as those based on *F*, and *R*- factors based on ALL data will be even larger.

### Fractional atomic coordinates and isotropic or equivalent isotropic displacement parameters (Å^2^)

**Table:**

	*x*	*y*	*z*	*U*_iso_*/*U*_eq_	Occ. (<1)
Sn1	0.57193 (3)	1.00258 (2)	0.124910 (18)	0.06021 (12)	
Cl1	1.19119 (17)	0.83857 (14)	0.33475 (12)	0.1064 (6)	
N1	0.6662 (4)	0.8684 (2)	0.1157 (2)	0.0487 (8)	
O1	0.7029 (4)	1.0206 (2)	0.2248 (2)	0.0767 (10)	
O2	0.4697 (4)	0.9344 (2)	0.0257 (2)	0.0687 (10)	
O3	0.4602 (5)	0.8138 (3)	−0.0595 (2)	0.0863 (12)	
C1	0.8139 (5)	0.9793 (3)	0.2452 (3)	0.0593 (12)	
C2	0.8952 (5)	1.0175 (4)	0.3079 (3)	0.0662 (14)	
H2	0.8713	1.0736	0.3320	0.079*	
C3	1.0080 (5)	0.9745 (4)	0.3343 (3)	0.0731 (16)	
H3	1.0598	1.0009	0.3762	0.088*	
C4	1.0458 (5)	0.8911 (4)	0.2988 (3)	0.0634 (13)	
C5	0.9727 (5)	0.8519 (4)	0.2370 (3)	0.0621 (12)	
H5	1.0000	0.7967	0.2128	0.075*	
C6	0.8562 (5)	0.8947 (3)	0.2100 (3)	0.0514 (11)	
C7	0.7776 (5)	0.8442 (3)	0.1488 (3)	0.0501 (11)	
H7	0.8119	0.7877	0.1314	0.060*	
C8	0.6029 (5)	0.8014 (3)	0.0581 (3)	0.0531 (11)	
H8	0.6717	0.7745	0.0220	0.064*	
C9	0.5027 (5)	0.8536 (4)	0.0029 (3)	0.0633 (13)	
C10	0.5359 (5)	0.7231 (3)	0.1087 (3)	0.0559 (11)	
H10	0.6024	0.7005	0.1487	0.067*	
C11	0.4186 (6)	0.7574 (4)	0.1604 (4)	0.0824 (16)	
H11A	0.3504	0.7800	0.1236	0.124*	
H11B	0.4475	0.8062	0.1968	0.124*	
H11C	0.3838	0.7075	0.1935	0.124*	
C12	0.4958 (6)	0.6414 (4)	0.0554 (4)	0.0749 (16)	
H12A	0.4561	0.5951	0.0915	0.090*	
H12B	0.4279	0.6614	0.0162	0.090*	
C13	0.6054 (9)	0.5975 (5)	0.0073 (5)	0.127 (3)	
H13A	0.6356	0.6391	−0.0356	0.191*	
H13B	0.5737	0.5417	−0.0182	0.191*	
H13C	0.6777	0.5835	0.0444	0.191*	
C14	0.4069 (6)	1.0251 (5)	0.2030 (5)	0.109 (2)	0.650 (10)
H14A	0.3707	0.9668	0.2222	0.131*	0.650 (10)
H14B	0.4324	1.0614	0.2515	0.131*	0.650 (10)
C15	0.3044 (11)	1.0769 (11)	0.1505 (7)	0.129 (4)	0.650 (10)
H15A	0.3501	1.1236	0.1180	0.154*	0.650 (10)
H15B	0.2643	1.0339	0.1113	0.154*	0.650 (10)
C16	0.1960 (11)	1.1223 (10)	0.1995 (8)	0.121 (4)	0.650 (10)
H16A	0.2284	1.1804	0.2215	0.145*	0.650 (10)
H16B	0.1725	1.0834	0.2467	0.145*	0.650 (10)
C17	0.0742 (12)	1.1397 (14)	0.1476 (10)	0.127 (5)	0.650 (10)
H17A	0.0058	1.1657	0.1823	0.191*	0.650 (10)
H17B	0.0435	1.0826	0.1240	0.191*	0.650 (10)
H17C	0.0954	1.1820	0.1032	0.191*	0.650 (10)
C14'	0.4069 (6)	1.0251 (5)	0.2030 (5)	0.109 (2)	0.350 (10)
H14C	0.4107	0.9760	0.2443	0.131*	0.350 (10)
H14D	0.4273	1.0814	0.2331	0.131*	0.350 (10)
C15'	0.2619 (12)	1.0331 (14)	0.1803 (17)	0.129 (4)	0.350 (10)
H15C	0.2061	1.0219	0.2289	0.154*	0.350 (10)
H15D	0.2386	0.9893	0.1368	0.154*	0.350 (10)
C16'	0.2453 (19)	1.1319 (14)	0.1492 (18)	0.121 (4)	0.350 (10)
H16C	0.2594	1.1742	0.1954	0.145*	0.350 (10)
H16D	0.3121	1.1446	0.1068	0.145*	0.350 (10)
C17'	0.108 (2)	1.148 (2)	0.112 (2)	0.127 (5)	0.350 (10)
H17D	0.0937	1.2130	0.1052	0.191*	0.350 (10)
H17E	0.0419	1.1234	0.1494	0.191*	0.350 (10)
H17F	0.1018	1.1179	0.0589	0.191*	0.350 (10)
C18	0.6505 (7)	1.1061 (4)	0.0462 (4)	0.0822 (17)	
H18A	0.5828	1.1232	0.0057	0.099*	
H18B	0.6693	1.1601	0.0800	0.099*	
C19	0.7719 (6)	1.0809 (4)	0.0003 (4)	0.0811 (17)	
H19A	0.7537	1.0276	−0.0346	0.097*	
H19B	0.8403	1.0637	0.0402	0.097*	
C20	0.8236 (8)	1.1589 (5)	−0.0545 (5)	0.110 (2)	
H20A	0.7520	1.1790	−0.0909	0.132*	
H20B	0.8465	1.2104	−0.0185	0.132*	
C21	0.9395 (8)	1.1372 (6)	−0.1067 (5)	0.131 (3)	
H21A	1.0188	1.1411	−0.0735	0.197*	
H21B	0.9447	1.1804	−0.1522	0.197*	
H21C	0.9307	1.0758	−0.1287	0.197*	

### Atomic displacement parameters (Å^2^)

**Table:**

	*U*^11^	*U*^22^	*U*^33^	*U*^12^	*U*^13^	*U*^23^
Sn1	0.0721 (2)	0.05157 (17)	0.05693 (18)	0.00367 (19)	−0.00498 (14)	−0.0011 (2)
Cl1	0.0716 (10)	0.1423 (15)	0.1053 (12)	0.0123 (11)	−0.0282 (9)	−0.0046 (11)
N1	0.058 (2)	0.0482 (18)	0.0403 (19)	0.0012 (16)	−0.0024 (18)	−0.0085 (16)
O1	0.090 (3)	0.065 (2)	0.075 (2)	0.017 (2)	−0.019 (2)	−0.0294 (17)
O2	0.088 (3)	0.060 (2)	0.059 (2)	−0.0046 (19)	−0.0222 (18)	0.0044 (16)
O3	0.128 (3)	0.075 (2)	0.056 (2)	−0.027 (2)	−0.030 (2)	−0.0001 (19)
C1	0.064 (3)	0.062 (3)	0.052 (2)	−0.003 (2)	0.004 (2)	−0.011 (2)
C2	0.073 (3)	0.069 (3)	0.057 (3)	−0.004 (3)	0.000 (2)	−0.026 (2)
C3	0.067 (3)	0.104 (5)	0.048 (2)	−0.022 (3)	−0.003 (2)	−0.008 (3)
C4	0.051 (3)	0.084 (4)	0.056 (3)	−0.003 (2)	−0.001 (2)	−0.001 (3)
C5	0.061 (3)	0.071 (3)	0.054 (3)	0.000 (2)	0.002 (2)	−0.009 (2)
C6	0.055 (3)	0.058 (3)	0.041 (2)	−0.006 (2)	0.006 (2)	−0.009 (2)
C7	0.061 (3)	0.045 (2)	0.044 (2)	−0.004 (2)	0.005 (2)	−0.0067 (18)
C8	0.061 (3)	0.052 (2)	0.047 (2)	−0.006 (2)	0.001 (2)	−0.0088 (19)
C9	0.075 (3)	0.069 (3)	0.046 (3)	−0.024 (3)	−0.013 (2)	0.010 (2)
C10	0.068 (3)	0.050 (2)	0.050 (3)	−0.008 (2)	−0.008 (2)	0.0027 (19)
C11	0.094 (4)	0.077 (3)	0.076 (3)	−0.009 (3)	0.013 (3)	0.012 (3)
C12	0.090 (4)	0.057 (3)	0.079 (4)	−0.016 (3)	−0.002 (3)	−0.004 (3)
C13	0.142 (6)	0.089 (4)	0.152 (6)	−0.018 (5)	0.027 (5)	−0.046 (5)
C14	0.088 (4)	0.122 (5)	0.116 (5)	0.033 (4)	0.019 (4)	−0.017 (4)
C15	0.096 (6)	0.147 (9)	0.142 (8)	0.004 (6)	0.031 (6)	−0.001 (7)
C16	0.120 (7)	0.129 (7)	0.114 (8)	0.023 (6)	0.012 (6)	0.016 (6)
C17	0.116 (7)	0.133 (6)	0.134 (10)	−0.002 (6)	−0.017 (7)	−0.006 (7)
C14'	0.088 (4)	0.122 (5)	0.116 (5)	0.033 (4)	0.019 (4)	−0.017 (4)
C15'	0.096 (6)	0.147 (9)	0.142 (8)	0.004 (6)	0.031 (6)	−0.001 (7)
C16'	0.120 (7)	0.129 (7)	0.114 (8)	0.023 (6)	0.012 (6)	0.016 (6)
C17'	0.116 (7)	0.133 (6)	0.134 (10)	−0.002 (6)	−0.017 (7)	−0.006 (7)
C18	0.111 (5)	0.049 (3)	0.087 (4)	−0.009 (3)	−0.004 (4)	0.007 (3)
C19	0.089 (4)	0.072 (4)	0.082 (4)	−0.006 (3)	−0.002 (3)	0.002 (3)
C20	0.122 (6)	0.099 (5)	0.108 (5)	−0.007 (5)	0.022 (5)	0.019 (4)
C21	0.133 (7)	0.132 (7)	0.129 (7)	−0.015 (5)	0.038 (6)	0.016 (5)

### Geometric parameters (Å, °)

**Table:**

Sn1—O1	2.083 (3)	C13—H13B	0.9600
Sn1—C14	2.100 (6)	C13—H13C	0.9600
Sn1—C18	2.110 (6)	C14—C15	1.526 (9)
Sn1—O2	2.130 (3)	C14—H14A	0.9700
Sn1—N1	2.169 (3)	C14—H14B	0.9700
Cl1—C4	1.745 (5)	C15—C16	1.493 (9)
N1—C7	1.287 (6)	C15—H15A	0.9700
N1—C8	1.481 (5)	C15—H15B	0.9700
O1—C1	1.308 (6)	C16—C17	1.500 (9)
O2—C9	1.271 (6)	C16—H16A	0.9700
O3—C9	1.227 (6)	C16—H16B	0.9700
C1—C2	1.406 (6)	C17—H17A	0.9600
C1—C6	1.415 (6)	C17—H17B	0.9600
C2—C3	1.362 (7)	C17—H17C	0.9600
C2—H2	0.9300	C15'—C16'	1.525 (10)
C3—C4	1.388 (7)	C15'—H15C	0.9700
C3—H3	0.9300	C15'—H15D	0.9700
C4—C5	1.355 (7)	C16'—C17'	1.518 (10)
C5—C6	1.394 (7)	C16'—H16C	0.9700
C5—H5	0.9300	C16'—H16D	0.9700
C6—C7	1.454 (6)	C17'—H17D	0.9600
C7—H7	0.9300	C17'—H17E	0.9600
C8—C9	1.538 (7)	C17'—H17F	0.9600
C8—C10	1.548 (6)	C18—C19	1.470 (9)
C8—H8	0.9800	C18—H18A	0.9700
C10—C12	1.513 (7)	C18—H18B	0.9700
C10—C11	1.523 (7)	C19—C20	1.520 (9)
C10—H10	0.9800	C19—H19A	0.9700
C11—H11A	0.9600	C19—H19B	0.9700
C11—H11B	0.9600	C20—C21	1.466 (10)
C11—H11C	0.9600	C20—H20A	0.9700
C12—C13	1.486 (9)	C20—H20B	0.9700
C12—H12A	0.9700	C21—H21A	0.9600
C12—H12B	0.9700	C21—H21B	0.9600
C13—H13A	0.9600	C21—H21C	0.9600
			
O1—Sn1—C14	91.5 (2)	C12—C13—H13C	109.5
O1—Sn1—C18	97.4 (2)	H13A—C13—H13C	109.5
C14—Sn1—C18	122.6 (3)	H13B—C13—H13C	109.5
O1—Sn1—O2	158.65 (13)	C15—C14—Sn1	106.6 (5)
C14—Sn1—O2	97.6 (2)	C15—C14—H14A	110.4
C18—Sn1—O2	93.9 (2)	Sn1—C14—H14A	110.4
O1—Sn1—N1	83.57 (13)	C15—C14—H14B	110.4
C14—Sn1—N1	121.7 (2)	Sn1—C14—H14B	110.4
C18—Sn1—N1	115.7 (2)	H14A—C14—H14B	108.6
O2—Sn1—N1	75.21 (13)	C16—C15—C14	115.0 (9)
C7—N1—C8	116.7 (4)	C16—C15—H15A	108.5
C7—N1—Sn1	126.7 (3)	C14—C15—H15A	108.5
C8—N1—Sn1	116.3 (3)	C16—C15—H15B	108.5
C1—O1—Sn1	132.3 (3)	C14—C15—H15B	108.5
C9—O2—Sn1	121.0 (3)	H15A—C15—H15B	107.5
O1—C1—C2	119.6 (4)	C15—C16—C17	112.4 (10)
O1—C1—C6	123.7 (4)	C15—C16—H16A	109.1
C2—C1—C6	116.7 (5)	C17—C16—H16A	109.1
C3—C2—C1	121.6 (5)	C15—C16—H16B	109.1
C3—C2—H2	119.2	C17—C16—H16B	109.1
C1—C2—H2	119.2	H16A—C16—H16B	107.9
C2—C3—C4	120.1 (5)	C16—C17—H17A	109.5
C2—C3—H3	120.0	C16—C17—H17B	109.5
C4—C3—H3	120.0	H17A—C17—H17B	109.5
C5—C4—C3	121.0 (5)	C16—C17—H17C	109.5
C5—C4—Cl1	120.7 (4)	H17A—C17—H17C	109.5
C3—C4—Cl1	118.4 (4)	H17B—C17—H17C	109.5
C4—C5—C6	119.6 (5)	C16'—C15'—H15C	110.8
C4—C5—H5	120.2	C16'—C15'—H15D	110.8
C6—C5—H5	120.2	H15C—C15'—H15D	108.9
C5—C6—C1	121.1 (4)	C17'—C16'—C15'	111.7 (13)
C5—C6—C7	116.1 (4)	C17'—C16'—H16C	109.3
C1—C6—C7	122.7 (4)	C15'—C16'—H16C	109.3
N1—C7—C6	127.7 (4)	C17'—C16'—H16D	109.3
N1—C7—H7	116.2	C15'—C16'—H16D	109.3
C6—C7—H7	116.2	H16C—C16'—H16D	107.9
N1—C8—C9	108.3 (4)	C16'—C17'—H17D	109.5
N1—C8—C10	110.1 (3)	C16'—C17'—H17E	109.5
C9—C8—C10	112.0 (4)	H17D—C17'—H17E	109.5
N1—C8—H8	108.8	C16'—C17'—H17F	109.5
C9—C8—H8	108.8	H17D—C17'—H17F	109.5
C10—C8—H8	108.8	H17E—C17'—H17F	109.5
O3—C9—O2	125.1 (5)	C19—C18—Sn1	115.5 (4)
O3—C9—C8	117.4 (5)	C19—C18—H18A	108.4
O2—C9—C8	117.4 (4)	Sn1—C18—H18A	108.4
C12—C10—C11	110.7 (4)	C19—C18—H18B	108.4
C12—C10—C8	113.3 (4)	Sn1—C18—H18B	108.4
C11—C10—C8	112.4 (4)	H18A—C18—H18B	107.5
C12—C10—H10	106.6	C18—C19—C20	112.7 (6)
C11—C10—H10	106.6	C18—C19—H19A	109.0
C8—C10—H10	106.6	C20—C19—H19A	109.0
C10—C11—H11A	109.5	C18—C19—H19B	109.0
C10—C11—H11B	109.5	C20—C19—H19B	109.0
H11A—C11—H11B	109.5	H19A—C19—H19B	107.8
C10—C11—H11C	109.5	C21—C20—C19	116.0 (7)
H11A—C11—H11C	109.5	C21—C20—H20A	108.3
H11B—C11—H11C	109.5	C19—C20—H20A	108.3
C13—C12—C10	115.4 (5)	C21—C20—H20B	108.3
C13—C12—H12A	108.4	C19—C20—H20B	108.3
C10—C12—H12A	108.4	H20A—C20—H20B	107.4
C13—C12—H12B	108.4	C20—C21—H21A	109.5
C10—C12—H12B	108.4	C20—C21—H21B	109.5
H12A—C12—H12B	107.5	H21A—C21—H21B	109.5
C12—C13—H13A	109.5	C20—C21—H21C	109.5
C12—C13—H13B	109.5	H21A—C21—H21C	109.5
H13A—C13—H13B	109.5	H21B—C21—H21C	109.5
			
O1—Sn1—N1—C7	−16.9 (4)	Sn1—N1—C7—C6	10.4 (7)
C14—Sn1—N1—C7	−104.7 (4)	C5—C6—C7—N1	178.8 (4)
C18—Sn1—N1—C7	78.3 (4)	C1—C6—C7—N1	3.1 (7)
O2—Sn1—N1—C7	165.4 (4)	C7—N1—C8—C9	−160.3 (4)
O1—Sn1—N1—C8	170.1 (3)	Sn1—N1—C8—C9	13.4 (4)
C14—Sn1—N1—C8	82.3 (4)	C7—N1—C8—C10	77.0 (5)
C18—Sn1—N1—C8	−94.7 (3)	Sn1—N1—C8—C10	−109.4 (4)
O2—Sn1—N1—C8	−7.5 (3)	Sn1—O2—C9—O3	−170.9 (4)
C14—Sn1—O1—C1	141.6 (5)	Sn1—O2—C9—C8	9.5 (6)
C18—Sn1—O1—C1	−95.3 (5)	N1—C8—C9—O3	165.7 (4)
O2—Sn1—O1—C1	26.2 (8)	C10—C8—C9—O3	−72.6 (6)
N1—Sn1—O1—C1	19.9 (5)	N1—C8—C9—O2	−14.7 (6)
O1—Sn1—O2—C9	−7.7 (7)	C10—C8—C9—O2	107.0 (5)
C14—Sn1—O2—C9	−122.1 (4)	N1—C8—C10—C12	−167.2 (4)
C18—Sn1—O2—C9	114.3 (4)	C9—C8—C10—C12	72.2 (5)
N1—Sn1—O2—C9	−1.2 (4)	N1—C8—C10—C11	66.3 (5)
Sn1—O1—C1—C2	167.9 (4)	C9—C8—C10—C11	−54.2 (5)
Sn1—O1—C1—C6	−14.7 (7)	C11—C10—C12—C13	−175.4 (6)
O1—C1—C2—C3	176.5 (5)	C8—C10—C12—C13	57.3 (7)
C6—C1—C2—C3	−1.0 (7)	O1—Sn1—C14—C15	144.2 (8)
C1—C2—C3—C4	0.5 (8)	C18—Sn1—C14—C15	44.4 (9)
C2—C3—C4—C5	0.8 (8)	O2—Sn1—C14—C15	−55.1 (8)
C2—C3—C4—Cl1	−180.0 (4)	N1—Sn1—C14—C15	−132.4 (8)
C3—C4—C5—C6	−1.4 (8)	Sn1—C14—C15—C16	−163.9 (11)
Cl1—C4—C5—C6	179.3 (4)	C14—C15—C16—C17	−157.9 (12)
C4—C5—C6—C1	0.9 (7)	O1—Sn1—C18—C19	79.1 (5)
C4—C5—C6—C7	−174.8 (4)	C14—Sn1—C18—C19	175.7 (4)
O1—C1—C6—C5	−177.1 (5)	O2—Sn1—C18—C19	−82.8 (5)
C2—C1—C6—C5	0.3 (7)	N1—Sn1—C18—C19	−7.3 (5)
O1—C1—C6—C7	−1.7 (7)	Sn1—C18—C19—C20	−179.5 (5)
C2—C1—C6—C7	175.7 (4)	C18—C19—C20—C21	−176.0 (6)
C8—N1—C7—C6	−176.7 (4)		
